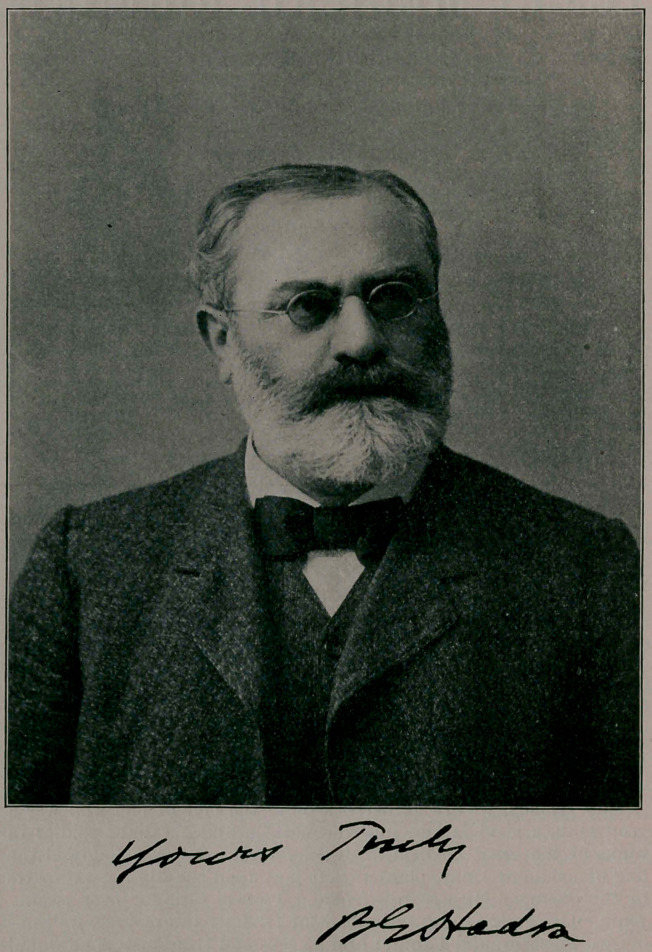# Death of Dr. Hadra

**Published:** 1903-07

**Authors:** 


### TEXAS MEDICAL JOURNAL.

AUSTIN, TEXAS.

A MONTHLY JOURNAL OF MEDICINE AND SURGERY.

EDITED AND PUBLISHED BY
F. E. DANIEL, M. D.

ASSOCIATE EDITORS:

WITTEN BOOTH RUSS, M. D., San Antonio, Texas.

P. M. PAYNE, M. D., Brownwood, Texas.

Published Monthly at Austin, Texas. Subscription price $1.00 a year in advance.

Eastern Representative: John Guy Monihan, St. Paul Building, 220 Broadway, New York City.

Official organ of the the West Texas Medical Association, the Houston District Medical Association, the Austin District Medical Society, the Brazos Valley Medical Association, the Galveston County Medical Society, and several others.

### DEATH OF DR. HADRA.

Dr. B. E. Hadra, of Dallas, one of the most famous physicians and surgeons of Texas, was found dead in his office in Dallas Sunday afternoon, July 12th (inst.). It is supposed that he died from the excessive heat of the day. He was very frail, and his friends had been anxious about him for many months past.

The remains were sent to Austin, and were interred in the family lot in the city cemetery. Dr. Hadra resided many years in Austin. The funeral was from the home of ex-Senator Walter Tips. and was attended by a large concourse of friends.

At Dallas, before the shipment of the remains, friends gathered at the family residence, and Dr. H. K. Leake, of that city, a warm friend of the deceased, paid a beautiful tribute to his memory.

The address delivered by Dr. Leake was as follows:

"My friends, we are gathered around the bier of one who was a good man, a good father, husband, citizen and a valiant soldier in ranks of the great medical army; a veteran who ever followed the flag of medicine—nay, planted that flag upon the highest ramparts of the enemy. He was my friend. I knew him for over twenty years. Oft, in the quiet retirement of friendly intercourse, we communed together, and I found him sincere and true, just as he would have been to any man in whom he placed his trust. He was ambitious, but humble; aggressive, but just and generous. He was a sensitive man, because he never indulged in raillery, nor violated the claims of propriety or courtesy, and expected that consideration which he always meted out to others. He was a fearless man in his work, which was based upon the highest scientific attainment and close application to his studies. His interest in his profession never

Yours Truly

B E Hadra failed him; to the day of his death he rivaled the younger men of the profession in zeal and research. He cared not for the sordid influence of money, nor groveled at the feet of the political machine; but he climbed the dizzy heights of fame only to realize the uncertainty of life and the illusory character of its emoluments.

"The boast of heraldry, the pomp of power,
And all that beauty, all that wealth e'er gave,
Await alike the inevitable hour—
The paths of glory lead but to the grave."

What lessons do this man's life and death teach the medical profession? "If they only knew," but we who do know the trials borne and the sacrifices made by the physician in the cause of suffering humanity and the disappointments that await each one of us at many points on our checkered careers, should be strongly cemented in the bonds of an unselfish and helpful brotherhood. We who do know that ofttimes we struggle against fate, nor can stay the hand of death, should pursue our calling free from strife and ostentation. Let us, then, realize our limitations; let the "white light of truth" guide our footsteps; let prudence and forbearance toward each other rule our actions; let devotion to morality and science be our guiding star, as it was that of our departed brother, who had no creed beside that of intelligent and unremitting application to his life's work and to his duties in all the relations of life. Let us be true to ourselves, and then we shall not be false to any man or creed.

My friend despised not religious creeds; he respected them, but his instincts were broad and unselfish. He preferred to live without them, but he lived and died with a pure and unsullied character, as a gentleman and physician. Surely, this were comprehensive enough for one whose instincts and education had molded him as the potter hath power over the clay, without its knowledge or consent.

We shall inscribe his name upon the memorial page of the historical annals of the medical profession of our State; we shall enshrine his virtues in our hearts; had he faults, we recall them not, for—

"Who knows the heart? 'Tis he alone decidedly can try us;
He knows each chord, its various tone; each string, its various bias.
Then at the balance let's be mute, we never can adjust it;
What's done we partly may compute, we know not what's resisted."

---

At the grave there was no religious ceremony, in accordance with the well-known wishes of the deceased, but Judge Julius Schutze delivered an eloquent and touching tribute to his life-long friend and compatriot, after which Dr. F. E. Daniel spoke a few brief words and touchingly referred to Dr. Hadra's life and services. Dr. Daniel said:

"It is my privilege and honor and a meloncholy pleasure to pay the tribute of a few poor words to the memory of him whom in life I loved and honored, and to testify to the high esteem in which Dr. Hadra was universally and justly held, not only by his colleagues, but by all who had the honor of his acquaintance. I knew him well; and for the brilliance of his genius, his splendid attainments as a physician, surgeon and general literary scholar I admired him, and for the excellence of his gentle heart and genial character I loved him. Dr. Hadra was no ordinary man. Indeed, he was a most extraordinary man, a rare man. Brilliant in his profession, his qualities amounted almost to genius. His strongest forte, perhaps, was the power of diagnosis. He seemed to have an intuitive knowledge of pathological conditions, and with remarkable insight he could detect what was wrong in a patient, and with great precision he could often relieve it by surgical means. His reputation as a surgeon was not local, nor confined to the State, nor to America; it was international. As a writer on those branches of medical and surgical science to which he devoted himself—operative surgery and gynecology—he was known and recognized as an authority throughout Europe and America. Moreover, he was a man of high literary attainments, and wrote several works of fiction which possessed real merit, but which he was too modest to publish. It was my privilege to see them in manuscript, but they have never been given to the public. I said he was 'too modest.' He was a very modest and unassuming man, as true merit ever is. It amounted at times to real embarrassment. When he was unanimously chosen by his colleagues to be President of the State Medical Association in April, 1899, at Waco, his embarrassment was almost painful, and he would have declined the honor but for the insistence of friends that he should not. He wanted to let the vice-president be president. The presidency of the State Medical Association means that the one so honored is recognized as the head of the profession. Honor was thrust upon him; it was never sought. He was appointed a member of the Board of Regents of the University of Texas and served four years. He filled the Chair of Surgery in the Medical Department of the University of Texas four or more years. At the time of his death he was one of the faculty of the Medical Department of the Southwestern University of Georgetown, at Dallas. Dr. Hadra was a Prussian. He was born near Breslau in 1842, and was in his sixty-first year. He served as surgeon major in the Prussian army in the Franco-Prussian war in 1870, and came to America shortly after that war. He had been thoroughly educated in the German universities, acquiring a good knowledge of the English language and its classical literature, and this knowledge he cultivated constantly, until he spoke and wrote it with a fluency and correctness attained seldom by any foreigner. Dr. Hadra was as guileless and gentle as a child and almost as improvident. He practiced surgery *con amore.* He never refused his services because there was no fee forthcoming; but he received many large fees, and he gave it away or wasted it. He was a somewhat eccentric man, and shrunk from notoriety. He wrapped himself around with a certain reserve, as to the general public, and few persons were permitted to break through it and get close to him. Those whom he admitted to the intimacy of his friendship, only knew and appreciated him. I was one of the favored few, and I say in all candor, there was not among my acquaintances one for whom I had a higher regard and real affection than Hadra—dear, delightful old Hadra! Yet, at the annual meetings of the State Medical Association he was a leader in thought and debate. He was recognized as one of our strong men, one of the pillars, one of the giants of the profession; and at the annual banquets no one contributed more than Hadra to the jollity, the merriment, the enjoyment of the occasion with his scintillating wit, his droll humor and his eloquence—forcible, not ornate—for he possessed a certain kind of eloquence that was most impressive. At our festive board for many long years there will be one vacant chair, around which will cluster the most sacred, the most pleasing, memories of dear Hadra. A long farewell, dear, gentle, delightful friend. Thy memory will be ever fresh and green. We beg of the bereaved family the privilege of sharing the grief that wrings their hearts, and of mingling with theirs our tears of sincere affection."

Dr. Hadra left a wife and five children: Dr. Fred Hadra, surgeon United States army; Ernest Hadra, druggist, at Dallas; Mrs. Eyssell, of Kansas City; James Hadra, and a young daughter, Ida.

### BIOGRAPHY.

Dr. Berthold Ernest Hadra was President of the Texas State Medical Association in 1899-1900, and presided at the Waco meeting in April, 1900. He was born in Prussia, near Breslau, in 1842; received his medical education in the universities of Breslau and Berlin, from which latter he graduated and where he passed his state examination.

He served as volunteer surgeon in the war against Austria (1866), and afterwards entered the Prussian army service.

In 1872 he immigrated to Texas, where he has since resided. He practiced his profession in Austin, Galveston and San Antonio. He was a member of the Board of Regents of the University of Texas; held the Chair of Surgery in the old Texas Medical College, and was health officer of San Antonio. His contributions to medical literature are numerous. Aside from a monograph on "Injuries of the Pelvic Floor," he was the first one to devise conservative surgical treatment in place of oöphorectomy, the so-called liberation of the pelvic organs. He was also the first one to propose total eventration of the contents and thorough washing and draining of the abdominal cavity in diffuse peritonitis. Repair of cystocele, perineum, etc., were frequent subjects of papers. To the surgery of the spine he contributed by adding wiring of the vertebræ. He has written also on the surgical treatment of epilepsy. To these many other original contributions, frequently quoted in international literature, may be added, such, for instance, as his paper on the open treatment of torticollis, on non-malignant tumors of the omentum, on relapsing appendicitis, on intestinal and gastric operations, etc.

## Figures and Tables

**Figure f1:**